# A Zinc Ointment for Varicose Ulcers

**Published:** 1886-10

**Authors:** 


A ZINC OINTMENT FOR VARICOSE ULCERS.—

| | | |
|---|---|---|
| ℞ | Zinci oxidi, - - - - | ʒ iiss. |
| | Gelatin, - - - - - | ʒ iiss. |
| | Glycerini, - - - - | ℥ i⅓. |
| | Aquæ, - - - - - | ℥ i⅓. |

Add the gelatin to three-fourths of the quantity of water and glycerin; when it is thoroughly dissolved, add the oxide of zinc, previously mixed with the remainder of the glycerin. Apply on a piece of cloth.

---

FRIEDREICH, of Heidelberg, reccommends cauterization of the clitoris for the cure of obstinate cases of hysteria. Nitrate of silver is the caustic employed. He has thus treated eight cases, all of whom were promptly cured after three or four cauterizations repeated at intervals of from three to ten days. Many years ago Kaker and Bronvon recommended ablation of the clitoris in cases of severe hysteria, claiming that the operation was always attended with good results.—*Revist Med. de Sevilla.*

---

Dr. AUSTINE BROWN, of Sioux Falls, has employed bromine, with unvarying success in seventy-five cases of ivy poisoning, using the following formula:

| | | | |
|---|---|---|---|
| ℞ | Bromine, - - - - | gtt. x-xx. | |
| | Ol. olivæ sen, - - - - | | |
| | Ol. Amygd. dulc *aa*, - - - - | ℥ j. | M. |

Apply freely, four times daily. Wash with warm water and castile soap twice daily.

---

BILE IN URINE.—Agitate the suspected urine with a few drops of chloroform in a test tube; if bile be present, the chloroform becomes turbid, acquiring a yellowish hue, the depth of which is proportionate to the amount of bile present. No bile being present the liquid remains limpid. Very minute quantities may be detected. When the cause of jaundice has been removed, salicylic acid is the most potent remedy in eliminating the bile pigment from the blood.

A Russian surgeon, Bruhns, publishes an article showing the danger of circumcision, as practised by the Jews. He mentions seven cases which resulted unfortunately, and claims that syphilis has been communicated by the careless methods of the Rabbi operator. Bruhns recommends that only physicians be allowed to perform this surgico-religious operation, and as the Jews are a persecuted people in Russia, it is quite probable that the State will interfere.

---

Philipowicz recommends puncturing the spleen with a hypodermic needle and drawing from its substance a syringeful of blood, for the diagnosis of typhoid fever. The bacteria thus obtained are cultivated in gelatine, and if they are the bacilli of typhoid, they may be recognized by their physiological and morphological without peculiarities. This operation has been performed twenty times any untoward results.

---

A Strange Verdict.—A verdict of $5,000 damages was awarded by a jury against a Buffalo physician for malpractice (?) in causing the death of a man who was so much intoxicated that he could not walk and fell from a street car, fracturing his fibula. The doctor gave two-thirds of a grain of morphine hypodermically in two doses. As the man died the jury attributed his death to the morphine.

---

In 1883 compulsory vaccination was abolished in Zurich, Switzerland. In 1882, there were no deaths from small-pox; in 1883, in a 1,000 deaths two were from small-pox; in 1884, there was three in a 1,000; in 1885, there were seventeen in a 1,000, and in the first quarter of 1886 there were eighty-five in a 1,000. These facts are eloquent in favor of compulsory vaccination.

---

Dr. W. Skinner, who formerly resided in Western New York, and is now practicing in Paris, France, publishes in *Le Progres Medical* an article entitled, "*Lymphangite et Sublime*," in which he shows that erysipelatous cellulitis, lymphangitis and erysipelas may be rapidly cured by the application of compresses wet with a 2 to 1,000 solution of bichloride of mercury.

